# Attenuation of Vaccinia Tian Tan Strain by Removal of Viral TC7L-TK2L and TA35R Genes

**DOI:** 10.1371/journal.pone.0031979

**Published:** 2012-02-21

**Authors:** Shifu Kan, Yuhang Wang, Lili Sun, Peng Jia, Yanxin Qi, Jiaqiang Su, Lei Liu, Guohua Yang, Liming Liu, Zhuoyue Wang, Jinhui Wang, Guangchen Liu, Ningyi Jin, Xiao Li, Zhuang Ding

**Affiliations:** 1 College of Animal Science and Veterinary Medicine, Jilin University, Jilin, People's Republic of China; 2 Genetic Engineering Laboratory of PLA, Academy of Military Medical Sciences of PLA, Jilin, People's Republic of China; 3 Department of Head and Neck Surgery, Tumor Hospital of Jilin Province, Jilin, People's Republic of China; 4 Animal & Plant Inspection and Quarantine Technology Center, Shenzhen Entry-Exit Inspection and Quarantine Bureau, Guangdong, People's Republic of China; 5 Norman Bethune School of Medical Science, Jilin University, Jilin, People's Republic of China; St. Louis University, United States of America

## Abstract

Vaccinia Tian Tan (VTT) was attenuated by deletion of the TC7L-TK2L and TA35R genes to generate MVTT3. The mutant was generated by replacing the open reading frames by a gene encoding enhanced green fluorescent protein (EGFP) flanked by loxP sites. Viruses expressing EGFP were then screened for and purified by serial plaque formation. In a second step the marker EGFP gene was removed by transfecting cells with a plasmid encoding cre recombinase and selecting for viruses that had lost the EGFP phenotype. The MVTT3 mutant was shown to be avirulent and immunogenic. These results support the conclusion that TC7L-TK2L and TA35R deletion mutants can be used as safe viral vectors or as platform for vaccines.

## Introduction

Vaccinia virus Tian Tan (VTT) strain has been studied for use as a live viral vector for infectious diseases or cancer therapy [1]. The VTT viral vector has been evaluated for vaccine development [2], [3], [4], but has issues relating to mild complications [5], [6], [7]. Therefore, the use, efficacy, and safety of the VTT strain requires re-evaluation [8], [9], [10]. Since the strain remains lethal to mice after intracranial inoculation, its use as a vaccine vector for humans is limited [5], and further attenuation of VTT will be necessary for its development as a useful vaccine vector.

Researchers are increasingly utilizing conditional gene manipulation strategies that allow deletion of a gene of interest [11]. One such approach is cumulative site-specific gene integration using the Cre-loxP recombination system. Among the site-specific gene recombination systems, the Cre-loxP system has been well studied and widely used for site-specific recombination in animal cells and functional analysis of genes [12], [13]. Cre recombinase recognizes a specific 34 bp loxP target sequence and catalyzes site-specific and irreversible cleavage of DNA segments flanked by unique loxP sequences. The enzyme catalyzes deletion, inversion, and exchange reactions depending on the number and direction of loxP sites inserted. In contrast to classical *in vivo* knockout strategies, which result in complete deletion of gene function in the whole organism, this conditional gene-targeting technology involving the recombinase-mediated cassette enables cell type-specific deletion of genes by driving the expression of Cre recombinase under the control of a cell type-specific promoter [14].

In the present study, we constructed a modified VTT genome (MVTT3) by deleting both TC7L-TK2L and TA35R genes, which resulted in reduced virulence. Deletion of small DNA fragments (3–25 nucleotides) are common in poxviruses [15]. Tartaglia et al deleted the C7L to K2L region (12ORFs) in the Copenhagen strain of vaccinia virus and reported phenotypic attenuation [16]. Rachel L. Roper showed A35R has little homology to any protein outside of poxviruses, suggesting a novel virulence mechanism [17]. Here, we examine the mutant virus which removed both TC7L-TK2L (15,262–25,450) including TC7L, TC6L, TC5L, TC4L, TC3L, TC2L, TC1L, TN1L, TN2L, TM1L, TM2L, TK1L, and TK2L and a single open reading frame TA35R (138,881–139,570) from vaccinia Tian Tan strain in relation to its virulence *in vitro* and *in vivo* [18], [19].

## Materials and Methods

### Cells, viruses and animals

BHK-21 hamster kidney cells, PK(15) porcine kidney cells, HeLa human cervical adenocarcinoma epithelial cells, Madin-Darby canine kidney (MDCK) epithelial cells, and Vero African green monkey kidney cells were obtained from the China Center for Type Culture Collection. The BHK-21 and PK(15) cells were cultured in modified Eagle's medium (MEM). HeLa and MDCK cells were cultured in Dulbecco's MEM. The Vero cells were cultured in RPMI 1640. Culture media (Invitrogen, Beijing, China) for the HeLa, MDCK, and Vero cells were supplemented with 10% fetal bovine serum (FBS; Hyclone, Beijing, China), 100 units/ml benzyl penicillin, and 100 µg/ml streptomycin sulfate. Media for the BHK-21 and PK(15) cells were supplemented with 5% FBS, 100 units/ml benzyl penicillin, and 100 µg/ml streptomycin sulfate. The parental vaccinia Tian Tan (GenBank accession no. AF095689) strain was obtained from the Institute of Virology at the Chinese Center for Disease Control and Prevention. Three- to five-week-old female BALB/c mice and female New Zealand white rabbits were purchased from the Experimental Animal Center of the Academy of Military Medical Sciences of China. The animal experimental protocols were approved by the Institutional Animal Care and Use Committee (IACUC) of the Chinese Academy of Military Medical Science, Changchun, China (10ZDGG007). All surgery was performed under sodium pentobarbital anesthesia, and all efforts were made to minimize suffering.

### Construction of VTT transfer vectors pSK-PTC7L-TK2L-EGFP and pSK-PTA35R-EGFP

The plasmid pVAX1-Cre, a derivative of pVAX1, was provided by Xiaoming Xia. Standard gene synthesis techniques were used to construct shuttle plasmid pSK-PTC7L-TK2L-EGFP and pSK-PTA35R-EGFP. A DNA fragment containing TC7L–loxP–PE/L–EGFP–loxP–TK2L sites was constructed by Shanghai Generay Biotech Co., Ltd. The PE/L–EGFP cassette was flanked by similarly oriented loxP sites. Replacement of the TC7L-TK2L gene by EGFP was confirmed using TC7LSP (5′-GTA CAT GAG TCT GAG TTC CTT G-3′) and TK2LASP (5′-ATC TGG CTA TTC TCC TTA GTT G-3′) as primers. The shuttle plasmid pSK-PTA35R-EGFP was constructed using the same strategy described above, with primers TA35RSP (5′-CAG CGT GAT TCT TAC CAG ATA TT-3′) and TA35RASP (5′-TGT TGC GAG CAT TAC TGC GTT TA-3′). The sequence flanked by the TC7L-TK2L gene was amplified to confirm the deletion of EGFP using TC7LSP1 (5′-CTT TGT GTA TCA TAT TCG TCC C-3′) and TK2LASP1 (5′- AAT TAG CGT CTC GTT TCA GAC T-3′) as primers. The sequence flanked by the TA35R gene was amplified to confirm the deletion of EGFP, using primers TA35RSP1 (5′- ACG AAT TAC ATT TCT TGT T-3′) and TA35RASP1 (5′- GCT ATG ATA TCT CTG GCT A-3′).

### Construction of MVTT1

Homologous recombination resulting in TC7L-TK2L replaced by the EGFP open reading frame (ORF) generated recombinant MVTT1-EGFP. This was accomplished by infecting 80% confluent BHK-21 cells with an input multiplicity of infection (MOI) of 0.01 PFU VTT per cell [20]. At 2 h post-infection, the cells were transfected with 2–3 µg of pSK-PTC7L-TK2L-EGFP in 10 µl of Lipofectamine 2000 (Invitrogen). Forty-eight hours after co-transfection at 37°C, virus was released by three freeze–thaw cycles and used for further infection and selection. Fluorescing plaques i.e., expressing MVTT1-EGFP were picked under an inverted fluorescence microscope and purified by 5 cycles of plaque isolation. The purity of the grown mutant was verified by PCR amplification and sequencing of the gene region flanking and within the TC7L-TK2L. MVTT1-EGFP genomic DNA was harvested using a Genomic DNA Miniprep kit (Axygen Biosciences). The PCR was carried out using MVTT1-EGFP genomic DNA as template and the primers TC7LSP and TK2LASP through 30 cycles of 95°C for 5 min, 94°C for 30 s, 55°C for 45 s, and 72°C for 45 s with the last extension at 72°C for 10 min. The product was resolved by 1% agarose gel electrophoresis.

BHK-21 cells (1×10^4^) were infected with 100 PFU MVTT1-EGFP. At 2 h post-infection, the cells were transfected with shuttle plasmid pVAX1-Cre. Forty-eight hours after co-transfection, non-EGFP-expressing plaques were picked and isolated by five rounds of plaque purification. The MVTT1 was propagated in BHK-21 cells. PCR amplification was used to confirm the deletion of EGFP. The MVTT1 genomic DNA was extracted as template, the primers TC7LSP1 and TK2LASP1 through 30 cycles at 95°C for 5 min, 94°C for 30 s, 55°C for 45 s, and 72°C for 45 s with the last extension at 72°C for 10 min. The product was resolved by 1% agarose gel electrophoresis.

### Construction of MVTT2

Recombinant MVTT2 was constructed using the same strategy described above. The TA35R gene was knocked out successively by homologous recombination with pSK-PTA35R-EGFP, and removal of EGFP by the Cre-loxP system. The double deletions were confirmed using the same PCR amplifications as shown above.

### Construction of MVTT3

The MVTT1 strain was used in a second homologous recombination. A similar procedure was used to delete the TA35R ORF from the MVTT1 strain to form MVTT3. The double deletions were confirmed using the same PCR amplification as it was shown above.

### Genetic stability of the mutants

Monslayers of BHK-21 cells were infected with the three mutants at 100 PFU and serially passaged 10 times to evaluate their genetic stability [21]. PCR and oligonucleotides flanking the regions deleted were used to demonstrate consistent amplification of the corresponding regions.

### Cell viability assay

The test used 3-(4,5-dimethylthiazol-2-yl)-2,5-diphenyltetrazolium bromide (MTT; Sigma, St. Louis, MO). The BHK-21, MDCK, HeLa, Vero, and PK(15) cells were seeded in 96-well plates (1×10^4^ cells/well) 1 d before they were infected with various concentrations (0.05 MOI) of MVTT1, MVTT2, MVTT3, or VTT. The viability of the five cell lines were tested every 24 h by treating cells with 20 µl of MTT (5 mg/ml) and incubating for 4 h at 37°C. The culture medium was removed, and the formed crystals were dissolved by exposing the cells in each well to 150 µl dimethylsulfoxide for 10 min at 37°C. The absorbance of the suspension at 490 nm was measured by an enzyme-linked immunosorbent assay (ELISA) plate reader. Untreated cells were used as controls, and all samples were examined thrice. The cell survival rate was calculated using the following formula: 100×(Experiment absorbances)/(control absorbances) [22], [23].

### Crystal violet staining

To test the spread efficiently of mutants in different cells, plaques of the mutants were visualized by crystal violet staining. The three mutants MVTT1, MVTT2 and MVTT3 at 0.05 PFU/cell were added to the five cell lines in 12-well plates. The cells were overlaid with 1% agarose after 90 min of viral exposure and then incubated for another 48 h at 37°C. Cellular growth was stopped by removing the medium, and 0.5% crystal violet in 20% methanol was added to the cells for 10 minutes. CPE was visualized by removing the crystal violet.

### Titration of mutants

The five cell lines infected with VTT and the mutants, and the viruses were harvested after 96 h and titrated in BHK-21 cells. Titers of the mutants in different cell lines were determined by plaque assay and expressed as plaque-forming units (pfu)/ml virus suspension [24].

### Virulence assay

For the rabbit skin pathogenicity assay, three concentrations (10^6^, 10^7^, and 10^8^ pfu in 0.1 ml sterile PBS) of MVTT1, MVTT2, MVTT3, or VTT were injected intradermally on the shaved backs of rabbits. Each concentration was performed in three rabbits. The diameters of erythema resulting from inoculation were measured daily for 18 d.

Mutant virulence was evaluated by measuring the weight loss of inbred BALB/c mice after viral inoculation, according to a previously described method [25]. Groups of five-week-old mice were inoculated intranasally with 10^5^, 10^6^, or 10^7^ pfu of MVTT1, MVTT2, MVTT3, or VTT in 20 µl of sterile PBS. Body weights of each mouse were determined daily for 25 d [26], [27]. Uninfected mice were included as controls.

### Intracranial 50% lethal infectious dose (ICLD_50_) measurement

Twelve groups of 3-week-old female BALB/c mice (n = 6) were inoculated intracranially with 10^5^, 10^6^, or 10^7^ pfu of MVTT1, MVTT2, MVTT3, or VTT in 10 µl of sterile PBS and deaths were recorded daily for 14 d. ICLD_50_'s were calculated using the Reed–Muench method [28].

### Immunogenicity and efficacy *in vivo*

Groups of 6-week-old female BALB/c mice (n = 10) were injected intramuscularly (i.m.) with 5×10^4^ PFU/mouse of MVTT1, MVTT2, MVTT3 or VTT in 0.1 ml PBS, or mock infected with PBS. Three weeks later, sera were taken from caudal vein and each mouse received a booster of the same dose. Two weeks later, the animals were euthanized and samples of heat-inactivated sera were processed for mouse IL-2, IL-4, IL-10 or IFN-γ ELISA analysis using commercially kits (Groundwork Biotechnology Diagnosticate, USA).

To determine the *in vivo* efficacy of MVTT1, MVTT2 and MVTT3, 3-week-old female BALB/c mice (n = 10) were vaccinated intramuscularly as described above and 4 weeks later infected intranasally with 500×LD_50_ VTT strain [29]. Individual body weights were measured daily, and animals with a weight loss of >30% were killed. Uninfected mice were included as controls.

### Neutralization assay

Heat-inactivated mouse serum were serially diluted in twofold steps, mixed with the parental VTT virus strain at a concentration of 100 PFU per well, and transferred to a monolayer of BHK-21 cells. 96 h post-incubation, BHK-21 cells were inspected for cytopathic effects. IC_50_ were determined by the highest dilution of mouse serum that generated 50% viral plaque reduction and was calculated by the method of Reed and Muench [28].

## Results

### Generation of the mutants

**Figure 1:**
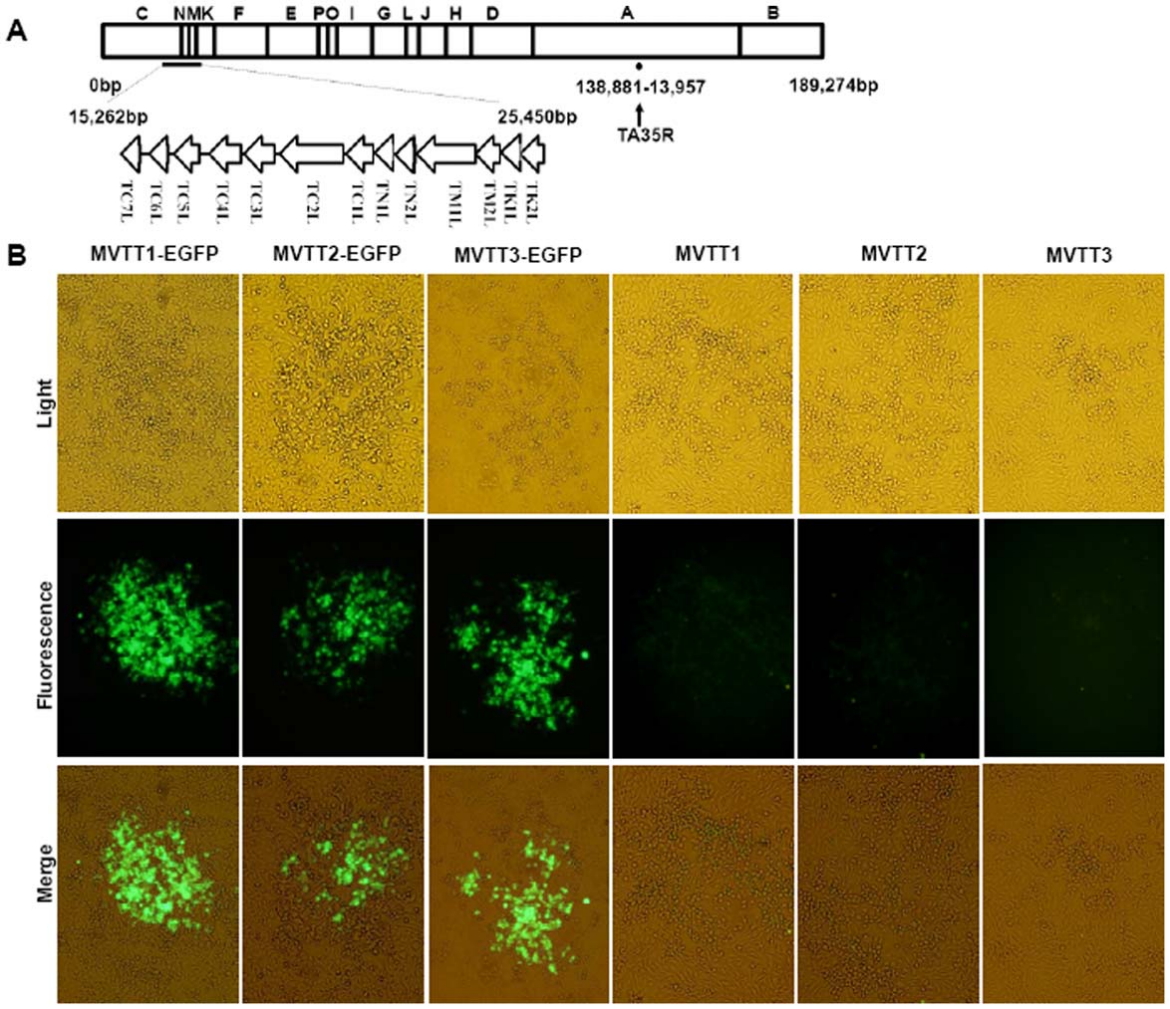
Schematic representation of the MVTT3 genome and identification of mutants. (A) The TC7L-TK2L and TA35R genomic deletions were found in the viral genome. (B) EGFP was expressed by mutants MVTT1-EGFP, MVTT2-EGFP, or MVTT3-EGFP in the BHK-21 cells. The virus-infected cells were visualized by isolated fluorescent plaque, which was recognized in the same visual fields. Non-fluorescent plaques appeared because of recombinant MVTT1, MVTT2, or MVTT3 in the BHK-21 cells. All photos were taken at 200× magnification.

**Figure 2:**
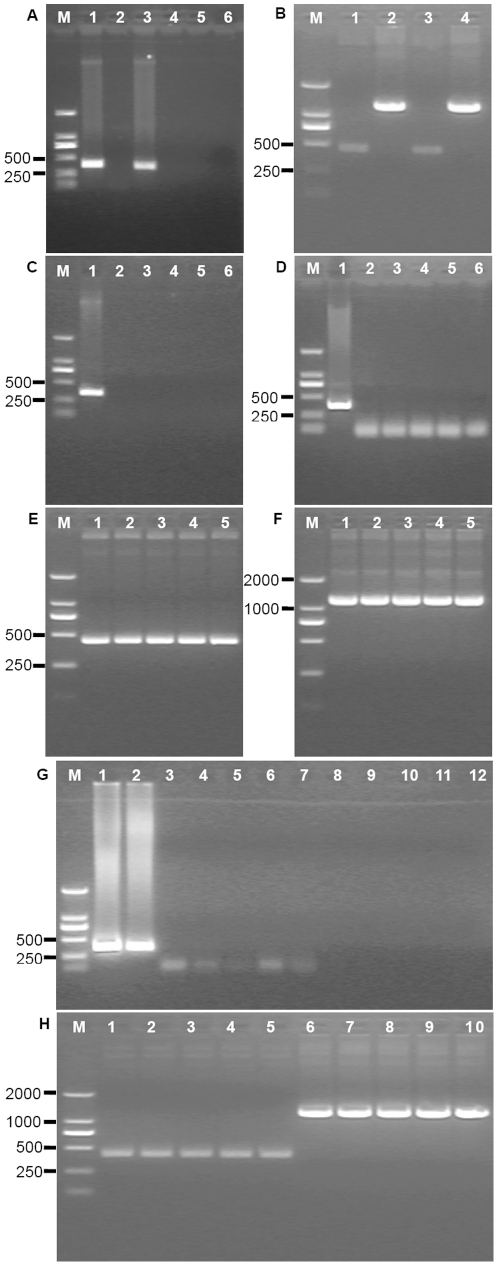
PCR analysis results of the TC7L-TK2L and TA35R genes of the isolated mutant. Diagnostic PCR was performed to identify the final mutant. The deletion of the TC7L-TK2L and TA35R genes was investigated by PCR 72 h after infection of BHK-21 cells with 0.01 MOI of wild-type VTT. Approximately 366 bp (TC7L-TK2L; A, lane 1) and 353 bp (TA35R; A, lane 3) were detected by ethidium bromide staining. In addition, the TC7L-TK2L and TA35R fragments were missing in the mutants MVTT1-EGFP (TC7L-TK2L; A, lane 2), MVTT2-EGFP (TA35R; A, lane 4), and MVTT3-EGFP (TC7L-TK2L and TA35R; A, lane 5 and lane 6). The genes 431 bp flanking the TC7L-TK2L regions (B, MVTT1, lane 1; and MVTT3, lane 3) and the genes 1142 bp flanking the TA35R regions (B, MVTT2, lane 2; and MVTT3, lane 4) were investigated to identify non-EGFP-expressing virus. Evaluation of the genetic stability in the BHK-21 cells after 2, 4, 6, 8 and 10 passages. TC7L-TK2L gene of MVTT1 (C, lanes 2–6), TA35R gene of MVTT2 (D, lane 2–6), TC7L-TK2L gene of MVTT3 (G, lanes 3–7), and TA35R gene of MVTT3 (G, lanes 8–12) produced negative results, comparing to positive PCR results. The genes of MVTT1 flanking the TC7L-TK2L regions (E), the genes of MVTT2 flanking the TA35R regions (F), and the genes of MVTT3 flanking the TC7L-TK2L regions (H, lanes 1–5) or TA35R regions (H, lanes 6–10) were detected correctly.

TC7L-TK2L and TA35R genes were deleted in VTT genome to generate three mutants (Fig. 1A). Plaques containing mutants were recognized by their fluorescence, and mutants were clonally purified in the BHK-21 cells by repeated plaque isolation (Fig. 1B). Fig. 2A showed that the double deletions 366 bp (9,357–9,732) of TC7L-TK2L and 353 bp (139,162–139,515) of TA35R were successful based on the PCR results for the plaque-purified EGFP virus.

Clones of purified non-fluorescent plaque in which TC7L-TK2L, TA35R, and both TC7L-TK2L and TA35R were deleted were identified (Fig. 1B). 431 bp (14,886–25,471) correct viral sequences flanking the deletion sites of TC7L-TK2L and 1142 bp (137,883–139,679) correct viral sequences flanking the deletion sites of TA35R were confirmed by nucleotide sequencing after removal of EGFP. Fig. 2B showed that the double deletions were successful based on the PCR results for the generation of three deletion viral mutants from VTT, namely, MVTT1, MVTT2 and MVTT3.

### Genetic stability of the mutants

The three mutants were added to cultures of BHK-21 cells passaged 10 times at 1 day intervals to determine their stability. The three genomic DNAs were then analyzed for the presence of TC7L-TK2L and TA35R. The mutant MVTT1 could not transcribe the gene within TC7L-TK2L after 2, 4, 6, 8, and 10 passages in BHK-21 cells, contrary to the result of VTT (Fig. 2C). Similar results were obtained with MVTT2 and MVTT3 (Fig. 2D and G). The correct 431 bp and 1142 bp viral sequences flanking the deletion sites of TC7L-TK2L and TA35R respectively were confirmed by nucleotide sequencing after 2, 4, 6, 8, and 10 passages. These findings indicate that the mutants were genetically stable after many passages (Fig. 2 E, F and H). This characteristic was crucial for eliminating the non-essential genes in developing a lower virulence vector for human use.

### Inhibition of cell growth by the mutants

**Figure 3:**
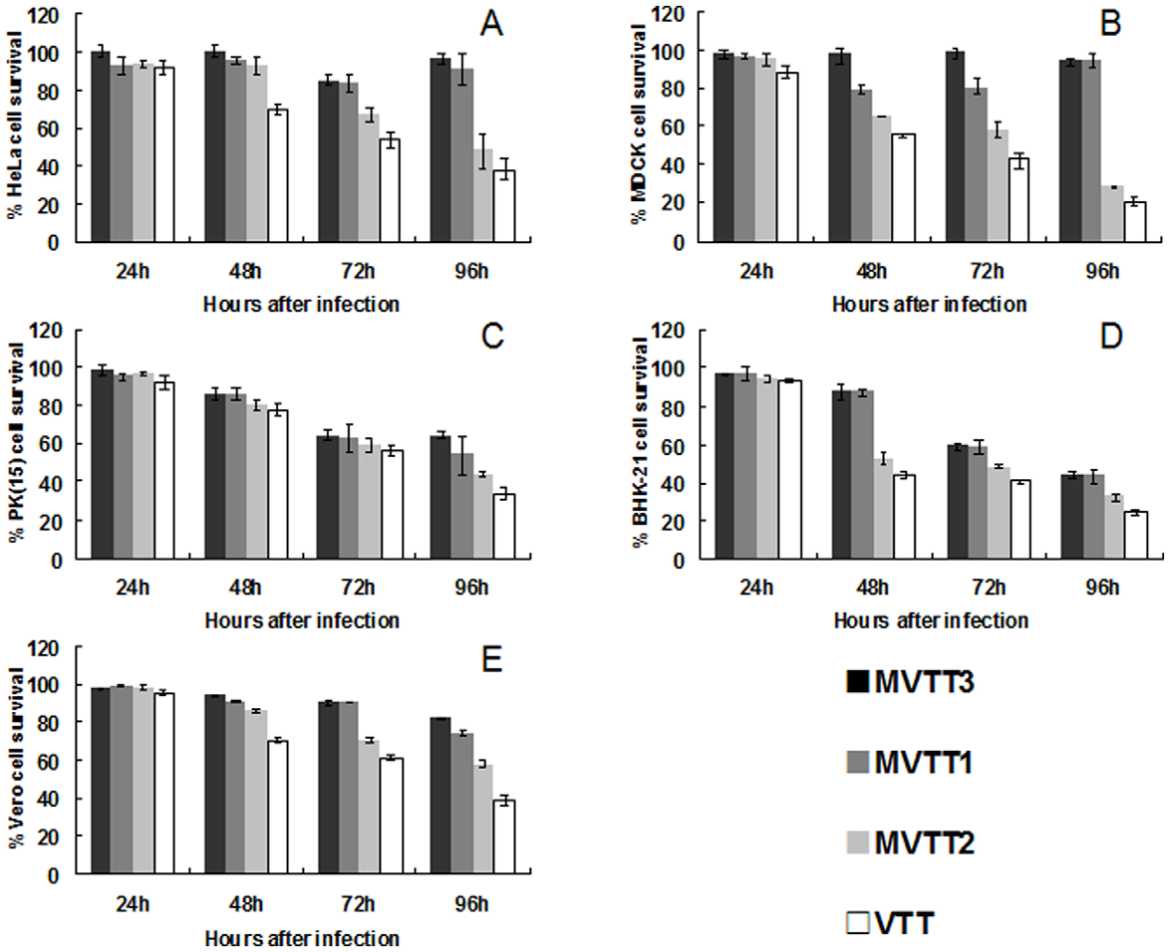
Assessment of growth of five cell lines infected with the mutants. Effects of MVTT1, MVTT2, MVTT3, or VTT (0.05 PFU/cell) and infection times on the cell viabilities of HeLa (A), MDCK (B), PK(15) (C), BHK-21 (D), and Vero (E). Cells were seeded in 96-well plates (1×10^4^ cells/well) one day before they were infected with 0.05 PFU/cell of MVTT1, MVTT2, MVTT3, or VTT. Cell viability was measured daily for 4 d by MTT colorimetric assay. All measurements were performed in triplicate. Data are presented as mean ± SD.

**Figure 4:**
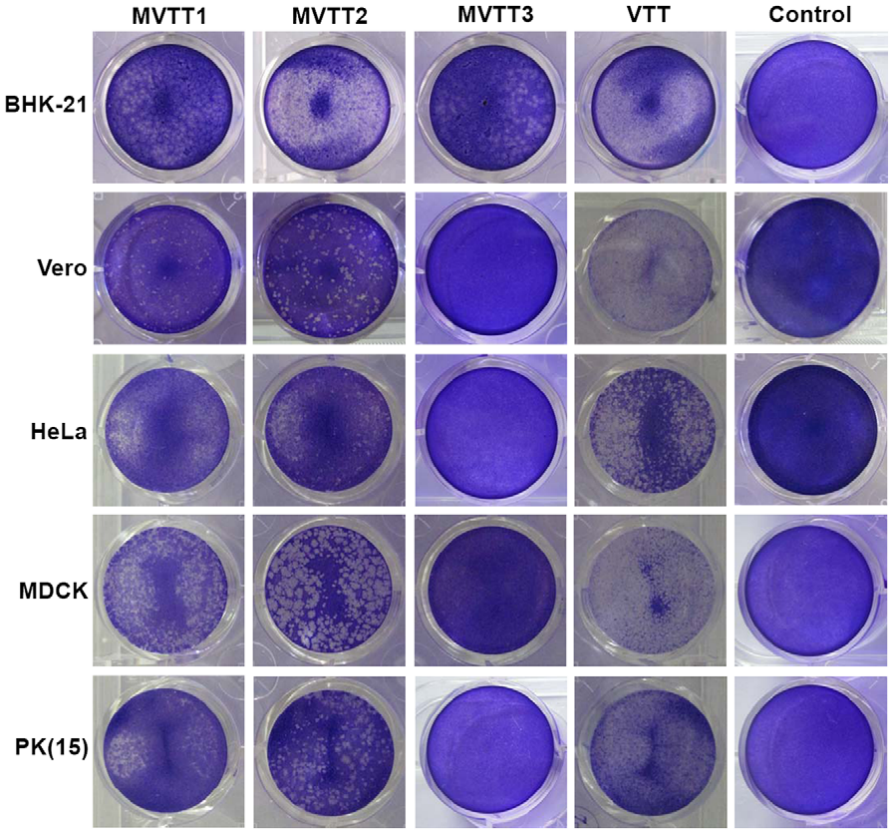
Plaque phenotypes formed by infection with mutant viruses. Confluent monolayers of PK(15), MDCK, HeLa, BHK-21 and Vero cells in 12-well plates were infected with 0.05 PFU/cell of MVTT1, MVTT2, MVTT3, or VTT viruses. The plates were incubated at 37°C for 2 d prior to staining with 0.1% crystal violet. The pathogenicity of MVTT1, MVTT2, and MVTT3 apparently decreased in all five cell lines (compared with VTT as the control).

The MTT colorimetric assay was performed to detect viabilities of the five cell lines after infection (Fig. 3). When cells were treated with the four viruses, MVTT3 had little effect on cell viability. In contrast, MVTT1, MVTT2 and VTT inhibited growth by 5%–50%, 45%–55% and 60%–80% after 96 h, respectively; growth of cells treated with VTT was eventually completely blocked. Therefore, the survival rates of the five cell lines infected with VTT were significantly lower than those infected with the mutants. The survival rate of cells infected with MVTT3 was higher than those infected with the other two mutants. The survival rate of cells infected with MVTT1 was higher than that of cells infected with MVTT2. Similar results were obtained by tests using crystal violet staining of the five cell lines infected with the mutants (Fig. 4).

### Mutants spread efficiently in five cell lines

The five mammalian cell lines were infected with 0.05 PFU/cell of each virus. Based on crystal violet staining, each cell type tested was found to be infected by VTT. Compared with VTT, MVTT3 apparently did not produce cytopathic effect in PK(15), MDCK, HeLa, and Vero cells (Fig. 4). The MVTT3 virus, however, displayed lower spread than MVTT1 (Fig. 4). The spread of MVTT1 evidently decreased than MVTT2, and MVTT2 displayed lower spread than VTT in all five cell lines, indicating that deletion of TC7L-TK2L further attenuated the virus (Fig. 4). These results also indicate that the loss of viral genomic fragments TC7L-TK2L or TA35R may not spread efficiently in tissue culture.

### Grown of mutants

**Figure 5:**
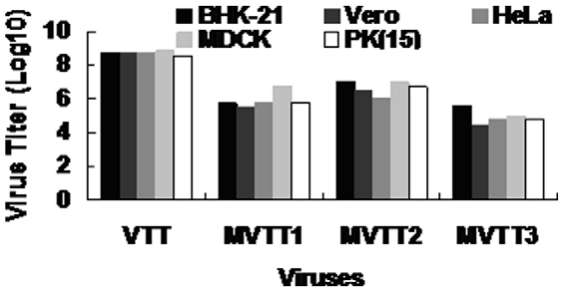
Virus titer of mutants in five cell lines tested. PK(15), MDCK, HeLa, BHK-21 and Vero cells infected with 0.05 MOI of VTT and the mutants, and then the viruses were harvested and titrated in BHK-21 cells. Virus titer was determined by measuring the plaque assays.

All three mutants proved capable of growing in the BHK-21 cells. The titration was shown in Fig. 5. The titers of the mutant viruses in each cell were lower than that of the parental virus. The titer of MVTT3 was lower than MVTT2 and MVTT1.

### Deletion of the TC7L-TK2L or TA35R gene attenuates VTT virulence in rabbits

**Figure 6:**
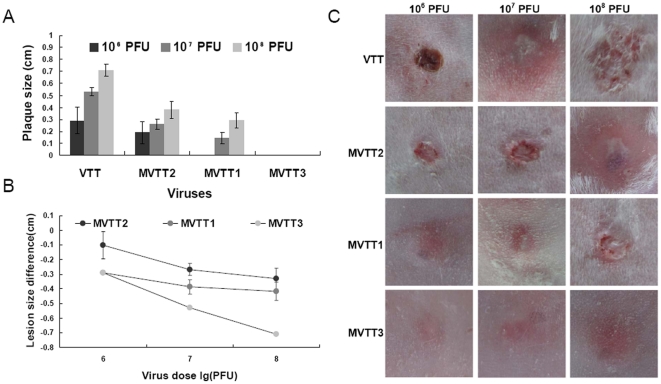
Characterization of virulence of strains intradermally injected in rabbits. Each rabbit was inoculated intradermally on the dorsal spine with a 10-fold dilution of MVTT1, MVTT2, MVTT3, or VTT (10^6^, 10^7^, and 10^8^ pfu). The central size diameters were recorded 18 d after inoculation. The diameters when erythemas reached their peak for each rabbit (A and C) are presented. The average difference is shown between lesion diameter produced by each recombinant and that by VTT (B). The data represents the mean ± SD of three lesions. The mean lesion sizes produced by MVTT1 and MVTT2 were significantly smaller than those formed by parental VTT. Lesions did not develop after inoculation with MVTT3 at the three doses.

Cutaneous lesion is a side effect of the virus inoculation; therefore, virulence was further assessed by intradermal inoculation on the rabbit dorsal spine. The lesion diameters were measured 18 d after inoculation. The diameters of the central lesions normally reached their peak on day 7. The diameters when the erythemas reached their peak are presented in Fig. 6A. The average differences in largest lesion diameter caused by each mutant were compared against that produced by VTT (Fig. 6B). The largest lesion size for each rabbit was shown in Fig. 6C. Rabbit skin remained intact when inoculated with varied doses of MVTT3, whereas the rabbit immunized with VTT developed a severe rash even when administered a dose of 10^6^ pfu. This was similar to the result of inoculation of 10^8^ pfu MVTT1. No significant difference between the lesions formed by MVTT1 and MVTT2 were observed (P>0.05). Recovery from skin lesions caused by VTT was slower than recovery caused by MVTT1 and MVTT2. The extent of recovery from lesions caused by MVTT1 was better than recovery from that caused by MVTT2.

### MVTT3 is safe for BALB/c mice

**Figure 7:**
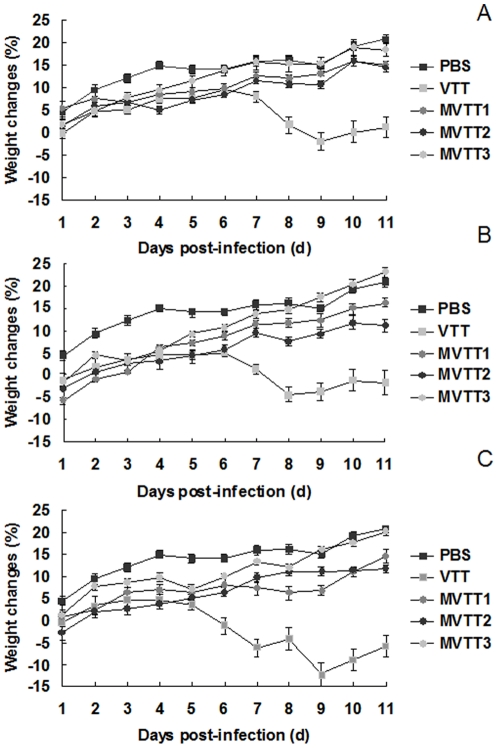
Virulence of MVTT1, MVTT2, and MVTT3 in mice after intranasal inoculations. Groups of mice (n = 6) were infected intranasally with different doses of MVTT1, MVTT2, MVTT3,VTT, or PBS on day 0, at doses of 10^5^ pfu (A), 10^6^ pfu (B), and 10^7^ pfu (C). The mean group weight was expressed as the percentage of the mean body weight change. The error bar indicates the standard deviation of each group. Each dose group of VTT and mutant showed a significant difference in weight change (P<0.05). No mice in the MVTT3 and PBS group lost weight or showed signs of illness.

Groups of six mice were infected with 10^5^, 10^6^, or 10^7^ PFU of MVTT1, MVTT2, and MVTT3 deletion mutants or VTT intranasally, and weight loss was recorded daily. As shown in Fig. 7, all mice survived during a period of 25 d. We show the first 11days after infection, which has significant differences between mutants group and VTT group. In contrast to the mice that received PBS, mice infected with MVTT1, MVTT2, or MVTT3 deletion mutants showed mild signs of illness, and mice in the MVTT3 group gained weight more quickly than did the mice in the other two groups. However, in contrast to VTT, there was an evident positive correlation between the dose of virus inoculation and the body weight loss. The VTT group had more severe signs of illness, and weight loss reached 12.19% on day 9. The lowest dose of 10^5^ pfu of VTT resulted in a significant body weight loss. The three mutants were likely attenuated by at least about 100-fold. The weight gain rate of MVTT3 was similar as PBS group. The weight gain rate of MVTT3 was higher than MVTT1 and MVTT2. These results suggest that MVTT1, MVTT2, and MVTT3 viruses were attenuated with respect to its parent VTT, and that the virulence of MVTT3 was less than that of the other strains. This experiment was repeated twice with similar results obtained.

### *In vivo* virulence of MVTT3

**Figure 8:**
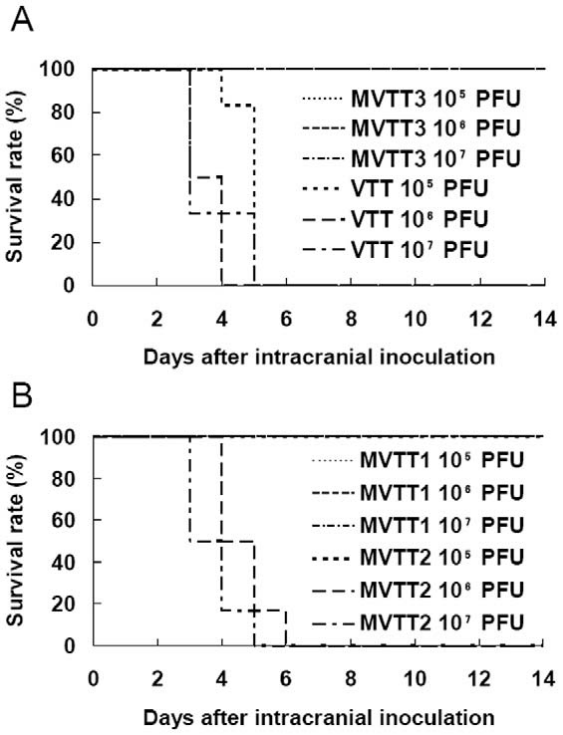
Protection from lethal challenge. Six 3-week-old mice in each dilution group were inoculated intracranially with 10^5^, 10^6^, and 10^7^ pfu of MVTT3 and VTT (A), and MVTT1 and MVTT2 (B). The survival rate of animals were observed for 14 d. All mice inoculated with MVTT1, MVTT3 and10^5^ pfu of MVTT2 survived. All mice infected with 10^6^ and 10^7^ pfu of MVTT2 and VTT died.

Six 3-week-old BALB/c mice were infected via the intracranial route to evaluate the neurovirulence of the three recombinant strains (Fig. 8). Since the ICLD_50_ of MVTT2 was 2.5×10^5^ pfu and that of VTT was 3.1×10^3^ PFU, MVTT2 was significantly attenuated by 80-fold. No mortality was observed in the mice infected with MVTT1 and MVTT3 although they were given the highest dose of 10^7^ pfu. These indicate that MVTT1 and MVTT3 were attenuated by at least 3,200-fold relative to the ICLD_50_ of VTT. The two mutants were essentially non-neurovirulent. These data indicate that the MVTT2 mutant exhibited attenuated but to a lesser degree than the MVTT1 and MVTT3 mutants. The results of the ICLD_50_ were consistent with those from the intranasal infection experiment. This experiment was repeated twice with similar results obtained.

### Immunogenicity of mutants

**Figure 9:**
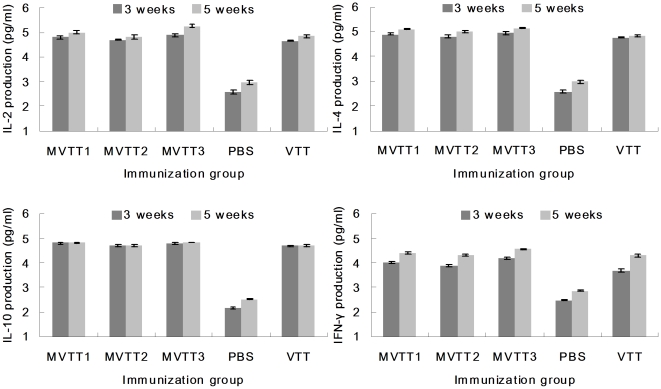
Immune responses induced by vaccination. Ten 6-week-old BALB/c mice received 5×10^4^ PFU MVTT1, MVTT2 or MVTT3 by intramuscular route and three weeks later received booster of the same dose in 0.1 ml of PBS. The serum were harvested on weeks 3 and 5 post infection (i.m.). Shown were induced IL-2, IL-4, IL-10 and IFN-γsystemic immune responses in the two vaccinated cohorts as measured by mouse ELISA kit analysis. All measurements were performed in triplicate. Data were presented as mean ± SD.

Delivery of MVTT1, MVTT2, MVTT3 and VTT elicited strong systemic immune responses induced by i.m. vaccination. IL-2, IL-4, IL-10 and IFN-γ responses, as measured by mouse ELISA, were already detectable using a spectrophotometer (Fig. 9). Mice were infected and on week three and five serum were harvested for IL-2, IL-4, IL-10 and IFN-γ production. The groups of MVTT1, MVTT2, MVTT3 and VTT, after twice administration, remained robust immune responses than PBS group. IL-10 responses was more difficult to induce. No significant response was seen after second immunization. For monitoring the production of IL-2, IL-4, and IFN-γ, responses were enhanced consecutively in twice i.m.-immunized. The difference among the mutants was not significant.

**Figure 10:**
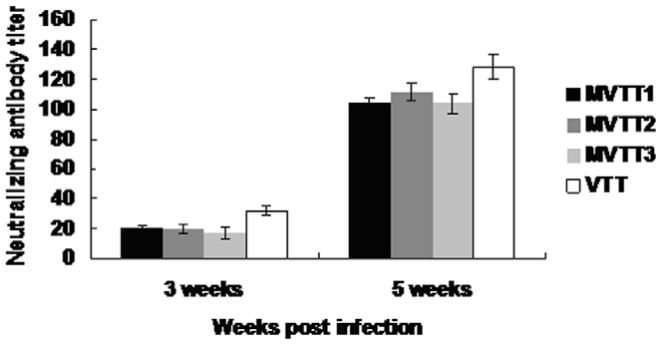
Neutralization antibody titer in murine sera. Murine sera collected at 3 and 5 weeks after infection. Neutralization antibody titer was calculated by determining the highest serum dilution to generated 50% viral plaque reduction. All measurements were performed in triplicate. Data were presented as mean ± SD.

Neutralizing antibody titer against the parental vector VTT was shown in Fig. 10. Immunization of each group induced systemic neutralizing antibody using the dose of 5×10^4^ PFU/mouse. The second immunization marked effects on level of neutralizing antibody. Neutralizing antibody titers of mutants are similar to those with the parental strain (P>0.05). Collectively, these results demonstrated that both mutants and VTT can induce robust systemic immune responses.

### Mutants protects mice from pathogenic VTT strain challenge

**Figure 11:**
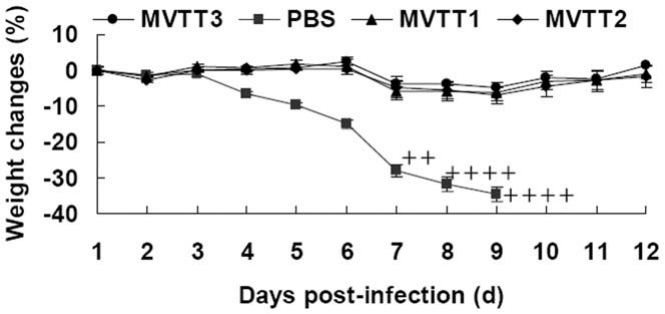
Protection of mice against pathogenic vaccinia VTT strain challenge. Groups of ten BALB/c mice (3-week-old) were vaccinated with 5×10^4^ PFU MVTT1, MVTT2 or MVTT3 via intramuscular routes. Mice received PBS were included as controls. Four weeks post-immunization, the mice were challenged intranasally with 500×LD_50_ VTT strain. Cross marks indicate the mice that were killed because of a 30% weight loss. Data show average percent change in pre-challenge weigh. The error bar indicates the standard deviation (SD) of animals from each group.

The protective immunogenicities of 5×10^4^ PFU MVTT1, MVTT2 and MVTT3 were determined by using a mouse model challenged with a highly pathogenic VTT strain. The mice immunized with MVTT1, MVTT2 and MVTT3 did not exhibit any significant differences in weight post challenge and no signs of illness. In contrast, beginning at the week of infection, all the mice in the PBS group clearly showed clinical signs of disseminated disease, such as ruffling fur and arched back. All mice immunized of MVTT1, MVTT2 or MVTT3 survived, whereas all sham-immunized mice were killed from 7 days because of a 30% weight loss (Fig. 11). All three mutants were equally effective in protection of mice against challenge with the VTT strain. These data support the conclusion that a VTT-based vaccine without TC7L-TK2L and TA35R genes would be a efficacious and immunogenic vaccine.

## Discussion

VTT was used as a vaccine against smallpox in China for millions of people, but remains neurovirulent in mice [5]. In this manuscript, we present the initial characterization of the TC7L-TK2L and TA35R deletion mutants in VTT to be avirulent. As previous study shows, the removal of the viral M1L-K2L genes (MVTT2-GFP) was less virulent than VTT for 340-fold by determining the ICLD_50_ value in mice [6]. Furthermore, the removal of the viral C2L to F3L genes (MVTT_ZCI_) was attenuated for 1000-fold [30]. However, MVTT1 was attenuated by at least 3,200-fold relative to the ICLD_50_ of VTT (Fig. 8), which was less virulent than MVTT2-GFP and MVTT_ZCI_. Others deleted C12L (10-fold) and A53R (4.6-fold) genes to be less virulent [31]. Base on VTT, this strategy enhance the attenuation potency. MVA [32] and NYVAC [33], as recombinant poxvirus vector, were highly attenuated to provide greater safety in humans. NYVAC was deleted of 18 specific open reading frames including the C7L-K1L genes [16]. No detectable induration or ulceration at the site of inoculation on rabbit skin [16]. This result was similar with MVTT3. Cutaneous lesion, more or less, could be seen after charged with MVTT1 or MVTT2 (Fig. 6). Not surprisingly, TA35R gene played a role in virulence [17]. We demonstrated that MVTT2 was attenuated by 80-fold. Base on MVTT1, the deletion of TA35R gene was one step closer to attenuation. Moreover, body weight changes were not significant, when mice were infected with the highest dose (Fig. 7). MVA is apathogenic in vivo, and virulence was clearly decreased in the same way [34]. Six major deletions emerged in the MVA genome [35]. Beside, C7L, C6L, N2L and K2L genes were deleted which were not associated with virulence in previous report. Because of the complexity of vaccinia viruses and the variety of combinational genes deletion reported, it was not yet possible to make a definitive statement about the correct gene of the most attenuation phenomenon. However, compared with attenuated strains such as the NYVAC, MVA and other VTT mutants, they all show attenuation in varying degrees.

The phenotypic changes observed in mutants were related to the loss of the TC7L-TK2L and TA35R genes in the VTT genome. As previous study shows, the loss of M1L-K2L genes reduced cell-to-cell spread including HeLa, PK(15), MDCK and Vero cells [6]. The loss of C2L to F3L genes reduced cell-to-cell spread including MDCK and Vero cells [30]. Consistent with previous findings, the titers of mutants changed significantly, and cell-to-cell spread was reduced in vitro. Of the genes, K1L and C7L prevent eIF2a phosphorylation to inhibit cellular and viral protein synthesis in modified VACV Ankara (MVA) infection [36]. K2L gene encode a serine protease inhibitor that inhibits cell–cell fusion [37]. In short, the titers and spread efficiency of MVTT1 and MVTT3 were decreased (Fig. 4). Nevertheless, C2L and A35 gene were not required for viral replication *in vitro* [17], [38]. Pathology of MVTT2 infected cells was slighter than VTT group (Fig. 4).

Vaccine candidates not only had strongly diminished virulence, but also maintaining good immunogenicity. Both MVA and NYVAC gave roughly comparable levels of protection against pathogenic vaccinia [39], [40]. During the generation of the attenuated NYVAC and MVA strains, some of those immunomodulatory genes were deleted or disrupted and this might explain why both viruses were highly immunogenic. The immunogenicity of the highly attenuated NYVAC was enhanced by the insertion of the C7L gene [41]. While, others demonstrate that C7L and K1L antagonize eIF2a phosphorylation, prevent PKR activation and inhibit antiviral activities [36], [42]. Furthermore, deleting C6L gene in MVA could improve the immunogenicity. Few studies showed that N2L and K2L were associated with immunogenicity in previous report. We therefore removed TC7L-TK2L gene from VTT in hopes of identifying its immunogenicity. Here, we demonstrated mutants induced immune responses was equal to those induced by VTT immunization. Deletion of the A35 gene resulted in increased immunogenicity in previous studies [29], [43]. Our study shows deletion of TA35R gene couldn't decrease immunogenicity of Modified VTT stain, which could be alternative vaccine vector. MVTT3 won't decrease immunogenicity significantly by genome remodel. It would be important to determine whether or not MVTT3 could protect animals against pathogenic VTT strain challenge. MVTT3 protect mice effectively from the challenge of a massive dose of VTT, suggesting that removal of TC7L-TK2L and TA35R genes from vaccinia Tian Tan strain-based vaccines will keep their immunogenicity. Since MVTT3 may offer greater advantage for avirulent and immunogenic, this strain could be used as an alternative, safer viral vector and vaccine for infectious diseases and cancer.
